# Assessing physical activity in people with posttraumatic stress disorder: feasibility and concurrent validity of the International Physical Activity Questionnaire– short form and actigraph accelerometers

**DOI:** 10.1186/1756-0500-7-576

**Published:** 2014-08-27

**Authors:** Simon Rosenbaum, Anne Tiedemann, Catherine Sherrington, Hidde P van der Ploeg

**Affiliations:** Musculoskeletal Division, The George Institute for Global Health, The University of Sydney, Missenden Road, PO Box M201, Sydney, NSW 2050 Australia; St John of God Healthcare Richmond Hospital, 177 Grose Vale Road, North Richmond, NSW 2754 Australia; Department of Public and Occupational Health, EMGO Institute for Health and Care Research, VU University Medical Center, Amsterdam, The Netherlands

**Keywords:** Adults, Exercise, Mental health, Physical activity, Posttraumatic stress disorder

## Abstract

**Background:**

Posttraumatic stress disorder (PTSD) is reportedly associated with lower rates of physical activity participation despite the known benefits of regular physical activity for improving both mental and physical health. However, no studies have evaluated the validity or feasibility of assessing physical activity within this population resulting in uncertainty around the reported lower rates of physical activity participation. This study aimed to evaluate the feasibility and concurrent validity of the International Physical Activity Questionnaire-Short Form (IPAQ-SF) and the Actigraph accelerometer (an objective physical activity monitor) among inpatients with PTSD.

**Methods:**

Fifty-nine adult hospital inpatients with a Diagnostic Statistical Manual Mental Disorder-IV-TR diagnosis of primary PTSD (mean age = 49.9 years; 85% male) participated in the study. Participants were asked to wear an Actigraph accelerometer for seven consecutive days then complete the IPAQ-SF. The Spearman rho correlation coefficient compared the amount of moderate to vigorous physical activity (MVPA) measured with the Actigraph and the total physical activity reported in the IPAQ-SF.

**Results:**

Lower than expected compliance with wearing accelerometers (<4 days valid data) (n = 20) was found suggesting that the use of accelerometers within this population may not be feasible. Complete IPAQ-SF data were available for 45 participants (76%) indicating that this tool also has its limitations in this population. The Spearman rho was 0.46 (p = 0.01) for the 29 participants with four or more valid days of accelerometer data (as per literature standards) and available IPAQ-SF.

**Conclusion:**

The IPAQ-SF and the Actigraph accelerometer have limitations in people with PTSD but in those able to provide data, show correlations of a magnitude comparable to those observed in the general population. The development and testing of mental health specific tools may enhance measurement of physical activity in this population.

## Background

Post-traumatic stress disorder (PTSD) has been recently reconceptualised in the most recent edition of the Diagnostic and Statistical Manual of Mental Disorders (5th edition) within a new dedicated chapter on trauma and stress related disorders. Previously identified as an anxiety disorder, PTSD is categorised by symptoms of hyper-arousal, re-experiencing and avoidance. Lifetime prevalence rates of PTSD within the general population are estimated to be between 5-10% [1], with occupation-specific rates among combat veterans and police officers estimated to be as high as 17% and 19% respectively [2, 3].

Similar to other mental health conditions, people with PTSD have higher rates of metabolic disturbances and poor physical health compared to the general population with higher rates of coronary heart disease, diabetes and substance abuse [4]. People with PTSD are less likely to engage in regular physical activity compared with the general population and are also more likely to be nicotine dependent [5, 6].

Regular physical activity, or structured exercise, has been shown to have anti-depressant and anxiolytic qualities [7, 8], although no randomised controlled trials have evaluated the effects of structured physical activity on symptoms of PTSD [9, 10]. Preliminary reports suggest that increasing physical activity within PTSD treatment programs may be beneficial for improving outcomes [9] whilst simultaneously reducing the incidence of metabolic syndrome and cardiovascular disease [11, 12].

As physical activity programs become routinely incorporated into PTSD treatment facilities, it is vital that clinicians have reliable and valid measures of physical activity that are also feasible to collect. Common methods of physical activity assessment include self-report questionnaires and objective monitors such as accelerometers. The International Physical Activity Questionnaire- Short Form (IPAQ-SF) is a frequently used four question, self-report measure of physical activity participation during the previous seven-day period [13]. A 2011 systematic review of validation studies reporting the correlation between total physical activity, as measured by an accelerometer, and the IPAQ-SF within both clinical and general populations found r values ranging from 0.09 to 0.39, with a tendency for the IPAQ-SF to over-estimate physical activity [14]. Despite this relatively low correlation with an objective measure, the IPAQ-SF is quick to administer, can be self-completed, and has previously been used with other psychiatric populations [15, 16]. No specific validity evidence exists for the IPAQ-SF for use among people with PTSD, and no studies to date have utilised accelerometers within this clinical population. Given the relatively higher rates of occupation-related PTSD found in physically demanding roles such as police and military service compared to sedentary occupations, it is reasonable to question and therefore test the validity of a self-report questionnaire within this specific population. Furthermore accelerometers have been previously used in the assessment of patients with mental illness, with compliance to wearing the monitors reported as low as 45% in one sample of 55 participants with varying psychiatric diagnoses [17]. As such we also felt it was necessary to test the feasibility of using accelerometers in patients with PTSD.

The aim of this study was to a) evaluate the feasibility of the International Physical Activity Questionnaire-Short Form (IPAQ-SF) and the Actigraph accelerometer among inpatients with posttraumatic stress disorder (PTSD) and b) determine the concurrent validity of these two instruments.

## Methods

### Sample and procedures

Fifty-nine adult participants were recruited from the inpatient trauma program at St John of God Health Care, Richmond Hospital (Australia) between May 2011 and April 2012. All participants met DSM-IV diagnostic criteria for PTSD, were ambulatory and cognitively able to provide consent. Potential participants were excluded if they were pregnant or medically restricted from participating in physical activity due to significant physical injury or illness. Ethical approval was obtained for this study from St John of God Health Care’s Ethical Committee (REF: 412) and The University of Sydney Human Research Ethics Committee (Protocol No 14091). Written informed consent was obtained from all participants.

Demographic information including age, sex and general medical and psychiatric history was collected by means of a questionnaire. The Health of Our Nation Outcome Scale (HoNOS) [18] was used to assess illness severity with the aim of determining what effect, if any, the severity of symptoms had on the correlation between the accelerometer and IPAQ-SF. The HoNOS is a standardised assessment tool used throughout mental health settings [18], and although not specific to PTSD, the HoNOS consists of 12 general items, each scored from 0–4 resulting in total scores of between 0 and 48, with higher scores indicating a greater illness severity.

The Actigraph GT3X accelerometer (Actigraph LLC, Pensacola, FL, US) is a small electronic device the size of a small beeper worn by individuals to record levels of physical activity and sedentary behaviour. Participants were provided with an Actigraph GT3X accelerometer as well as written wearing instructions, and were asked to wear the accelerometer on the right hip for all waking hours during a 7-day period, except during water-based activities. Participants were instructed to wear the accelerometer if napping during daytime hours. The Actigraph output includes movement counts/ minute for the vertical, anterior-posterior, and mediolateral axes. The epoch was set at 1-second intervals. Accelerometer cut-points were defined as sedentary (<100 counts/minute), light activity (100–1951 counts/minute), moderate activity (1952–5724 counts/minute) and vigorous activity (>5725 counts/minute) [19, 20]. In order to maximise accelerometer wear time, nursing staff prompted participants at regular intervals throughout the day (during required nursing rounds) to wear the monitors, and a reminder notice was posted on the unit whiteboard.

Following the 7-day period, participants completed the IPAQ-SF. The IPAQ-SF assesses four domains of physical activity over the previous week, including vigorous activity (activities that make breathing much harder than normal), moderate activity (activities that make breathing somewhat harder than normal), walking and time spent sitting. Data from the IPAQ-SF were scored as per the IPAQ-SF scoring manual [13]. As recommended by the manual, questionnaires were excluded from the analysis if they contained erroneously high values (cases in which the sum total of all walking, moderate and vigorous time variables is greater than 960 minutes). Total physical activity (minutes per week), was calculated by combining the totals for the walking, moderate and vigorous categories.

### Statistical analyses

Participants were included in the statistical analyses if they recorded at least three valid days of accelerometer data. Analyses were conducted with both at least three, and at least four valid days. A valid day was defined as at least 10 hours of wear time, and periods of 60-minutes or more of consecutive zeros were considered as non-wear time. All analyses were performed with SPSS statistical software. The concurrent validity of the IPAQ-SF against the accelerometer was determined using Spearman rho correlation coefficients, calculated for both minutes per day of moderate to vigorous physical activity, and total sitting (IPAQ-SF) and sedentary time (accelerometer) per weekday. Bland-Altman plots with 95% limits of agreement were calculated as the measures of agreement between the instruments. The median HoNOS score (21) was used as the dichotomisation point to stratify participants according to illness severity, and t-tests were used to determine the impact of illness severity on accelerometer-recorded physical activity.

## Results

Anthropomorphic, demographic and diagnostic participant data are included in Table 1. The mean age of participants was 49.9 years (SD = 11.9, range = 23–71 years). Mean Body Mass Index (BMI; height^2^ (m)/ weight (kg)) was 30.2 (SD = 4.6), with 9% of participants being normal weight, 48% overweight, and 44% obese (class I or II obesity) [21] (mean weight = 94.4 kg (SD = 16.2)). Previous psychiatric admissions were recorded, with the majority of participants having no previous psychiatric admissions (n = 39, 66%). Current or past serving police duties were cited as the leading cause of trauma (n = 29, 49%), with the next largest contributor being defence personnel duties (n = 15, 25%), followed by motor-vehicle accidents (n = 4, 7%). Nine participants cited other causes such as assault, sexual assault and non-specified traumatic experiences. The cause of experienced trauma was mostly employment-related (85%) with a mean time to diagnosis from the traumatic event 2.7 years (SD = 4.3). The mean total HoNOS illness severity score was 19.9 (SD = 6.5).

**Table 1:** **Participant demographic information**

	Total (n = 59)	Excluded* (n = 10)	Included (n = 49)
Male gender, n(%)	50 (85%)	7 (70%)	43 (88%)
Age, mean (SD)	49 (12)	48 (12)	51 (12)
Body Mass Index (n = 46)			
18.5-24 (healthy)	4 (9%)	1 (16%)	3 (8%)
25-29 (overweight)	22 (48%)	2 (34%)	20 (50%)
30-39 (obese)	20 (43%)	3 (50%)	17 (42%)
Previous psychiatric admissions, n(%)			
None	39 (67%)	7 (70%)	32 (65%)
1-5	12 (20%)	0	12 (23%)
6-10	3 (5%)	2 (20%)	1 (3%)
>10	5 (8%)	1 (10%)	4 (9%)
Trauma context			
Police officer	29 (51%)	7 (70%)	22 (46%)
Defense	15 (26%)	3 (30%)	14 (28%)
Motor vehicle accident	13 (23%)	0	13 (26%)
Lower illness severity (HoNOS <21)#	23 (41%)	3 (30%)	21 (45%)
Higher illness severity (HoNOS > =21)#	33 (59%)	7 (70%)	26 (55%)
Years since diagnosis, mean (SD)	2.7 (4.3)	1.7 (2.5)	2.9 (4.6)
Current smoker	16 (27%)	5 (50%)	11 (22%)
Benzodiazepam use (sedative)	19 (32%)	4 (40%)	15 (31%)

*Excluded from analysis due to < 3 days of valid accelerometer data or missing IPAQ-SF.

#n = 56 due to missing data.

In total ten of the 59 participants (17%) recorded zero, one or two valid days of accelerometer data, whilst only eight (14%) recorded the full seven valid days. A further ten participants (17%) recorded three valid days, five (15%) recorded four valid days, nine (15%) recorded five valid days and 17 (29%) recorded six valid days. Literature standard is to only include participants with at least four valid days of data, but given the poor compliance we performed sensitivity analyses for both 3+ and 4+ valid days, which showed similar results (Table 2).

**Table 2:** **Spearman rho correlations between the actigraph accelerometer and the International Physical Activity Questionnaire-Short Form (IPAQ-SF)**

	N	Accelerometer (mins/day)		N	IPAQ-SF (mins/day)	Spearman’s rho correlation
Moderate-vigorous physical activity						
3+ valid days	49	Mean (SD)	40 (31)	37	43 (38)	0.45
		Median (25th% - 75th%)	35 (20–54)		30 (11–69)	
4+ valid days	39	Mean (SD)	42 (31)	29	45 (38)	0.46
		Median (25th% - 75th%)	36 (22–54)		30 (16–69)	
Sedentary time (mins/weekday)						
3+ valid days	38	Mean (SD) Median (25th% - 75th%)	444 (393–550) 474 (119)	34	600 (360–840) 611 (321)	0.33
4+ valid days	29	Mean (SD) Median (25th% - 75th%)	481 (110) 440 (410–536)	27	598 (321) 540 (360–840)	0.29

In total seven of 59 (12%) IPAQ-SFs were incomplete due to sudden or early discharge and eight of 59 (14%) reported values that were considered erroneously high (physical activity ≥16 hours per day) and were excluded from the analysis as advised in the IPAQ-SF scoring manual. Illness severity was not found to significantly impact compliance with either the accelerometer or the IPAQ-SF.

As shown in Table 2, the Spearman rho correlation between moderate and vigorous physical activity based on the accelerometer and total physical activity from the IPAQ-SF was 0.46 (95% confidence interval (95% CI) = 0.11 to 0.71, ≥four valid days) and 0.45 (95% CI = 0.15 to 0.68, ≥three valid days). The Spearman correlation between sitting time per weekday based on the IPAQ-SF and sedentary time per day as assessed with the accelerometer was 0.29 (95% CI = 0.15 to 0.68, ≥four valid days) and 0.33 (≥three valid days).

For participants with a greater illness severity as defined by a HoNOS score of ≥21, the Spearman correlation for physical activity between the accelerometer and the IPAQ-SF was 0.46 (95%CI = 0.02 to 0.75, ≥three valid days; n = 20) and 0.59 (95%CI = 0.09 to 0.85, ≥four valid days; n = 14).

For those with a lesser illness severity the correlation was 0.46 (95%CI = −0.05 to 0.78) (≥three valid days; n = 16) and 0.48 (95%CI = −0.07 to 0.81, ≥four valid days; n = 14) (Table 3).

**Table 3:** **Accelerometer assessed physical activity data stratified by illness severity; mean (SD)**

	Lesser illness severity (HoNOS ≥21) (n = 21)	Greater illness severity (HoNOS <21) (n = 26)	P-value ( ***t***-test)
Counts/ day	303697 (194674)	259940 (112820)	0.12 ns
Steps/ day	8563 (3996)	8562 (3237)	0.63 ns
Light activity (mins/day)	197 (42)	214 (48)	0.56 ns
Moderate activity (mins/day)	42 (28)	35 (25)	0.72 ns
Vigorous activity (mins/day)	5 (16)	2 (3)	0.05 ns
Wear time (mins/day)	784 (89)	809 (96)	0.5 ns
Sedentary time (mins/day)	540 (75)	559 (83)	0.65 ns

The Spearman correlation between sitting time based on the IPAQ-SF and accelerometer for those with a greater illness severity was −0.001 (95%CI = −0.41 to 0.41, ≥three valid days; n = 23) and 0.33 (95%CI = −0.16 to 0.69, ≥four valid days; n = 18). For participants with a lesser illness severity the correlation was 0.28 (95% CI = −0.19 to 0.64, ≥three valid days; n = 20) and 0.34 (95% CI = −0.17 to 0.71), ≥four valid days; n = 17).

**Figure 1:**
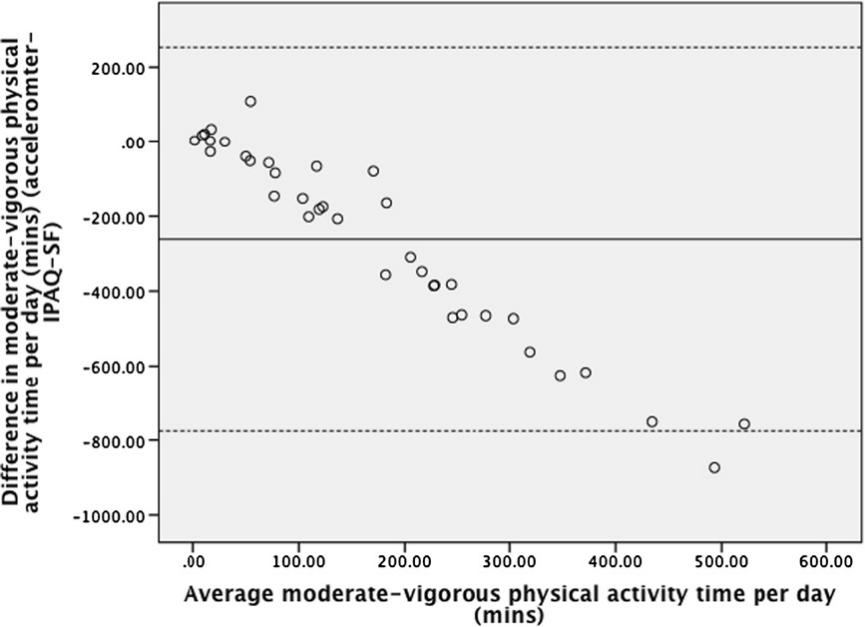
**Bland-Altman plot of agreement between accelerometer and self-reported moderate vigorous physical activity time.**

**Figure 2:**
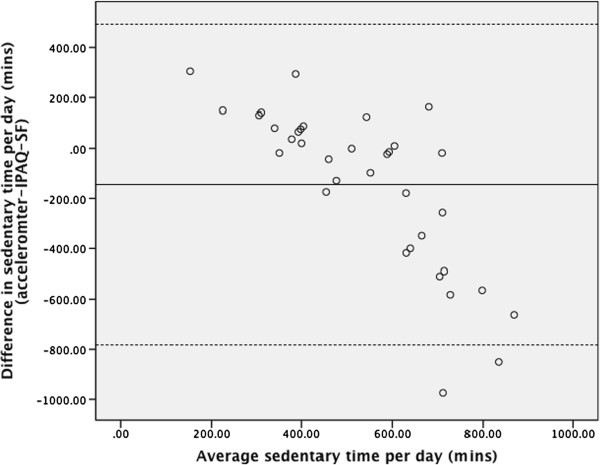
**Bland-Altman plot of agreement between accelerometer and self-reported sedentary time.**

The Bland-Altman plot for moderate-vigorous physical activity (Figure 1) indicates that the IPAQ-SF has poor agreement with the accelerometer, particularly as physical activity volume increases which is comparable to other studies investigating the relationship between accelerometers and the IPAQ [22]. The plot for sedentary time (Figure 2) shows poor agreement between the accelerometer and IPAQ-SF that worsens as mean sedentary time increases.

## Discussion and conclusions

This study found poorer than expected compliance with both measures of physical activity but found that, among those who provided data for both tools, the IPAQ-SF and Actigraph have similar correlations compared to the general population [22].

The high number of participants excluded from the final analysis due to invalid accelerometer data or erroneous IPAQ-SF questionnaire responses is an important finding. Contributing factors to poor accelerometer compliance may include a lack of motivation and fatigue, the location of the objective monitoring device (hip, wrist, ankle, waist), and external and internal motivators for wearing the monitors. Future studies utilising accelerometer measurements within inpatient PTSD populations may benefit from a multi-disciplinary approach to increasing compliance by utilising health professionals in contact with patients on a regular basis, such as nursing staff, psychologists and occupational therapists.

Of the 52 completed IPAQ-SF questionnaires, eight were excluded from the analysis due to erroneously high values. Future research with psychiatric inpatients may also benefit from using an interview to administer the IPAQ-SF to improve the accuracy of physical activity reporting. Nursing and allied health staff in regular contact with inpatients would be appropriately placed to administer such an interview. Given that 74% of participants in this study were from a police or defence background, it is possible that participants may have reported physical activity based on their pre-trauma levels of participation, in which physical activity and regular exercise would have been required for occupational purposes. Over reporting has previously been acknowledged as a key limitation of self-reported measures of physical activity [14].

Among those who provided data for both tools, the correlation between the IPAQ-SF and Actigraph was reasonably high (0.46) indicating acceptable concurrent validity for estimating the physical activity of inpatients with PTSD based on findings from similar studies in other populations [22]. This degree of correlation is comparable with that in the general population where values of up to 0.39 have been identified [14]. The missing data indicate that this value may not be transferrable to the general inpatient population with PTSD and suggest that the development of a tool specifically designed for people with mental health problems may be warranted.

Illness severity was not strongly associated with physical activity levels, or compliance with either the accelerometer or IPAQ-SF. Illness severity however did impact upon the strength of the relationship between time spent in sedentary behaviour and sitting time as per the IPAQ-SF. This has implications for practice, and highlights a need for greater physical activity interventions within psychiatric facilities, especially for those with a greater illness severity.

The high rate of excluded data is a significant limitation of this study, and serves to highlight the pragmatic limitations associated with assessing physical activity in PTSD. The relatively small number of female participants is another potential limitation, as differences in feasibility and compliance to either measure may exist between sexes that were unable to be assessed within this study. The IPAQ-SF asks participants about physical activity participation in bouts of at least ten-minutes only. This was not accounted for in the analysis of the accelerometer data, which we acknowledge as a potential limitation of this study.

Both the IPAQ-SF and accelerometers have limitations when used with inpatients with PTSD, including over-reporting and poor compliance to wearing objective monitors. The quick administration time and moderate correlation with an objective measure of physical activity are strengths of the IPAQ-SF, however further research is needed to identify optimal approaches to measurement of physical activity in people with mental health problems [16]. The development and testing of mental health specific tools may enhance measurement of physical activity in this population.
